# Reading man flap in four dogs: a case series

**DOI:** 10.1186/s12917-023-03723-z

**Published:** 2023-09-15

**Authors:** Desiree Siegelmayer, Gabriele Gradner

**Affiliations:** 1 University Clinic for Companion Animals of Vienna, Veterinärplatz 1, 1210 Vienna, Austria; 2 Diplomate of European College of Veterinary Surgeons, University Clinic for Companion Animals of Vienna, Veterinärplatz 1, 1210 Vienna, Austria

**Keywords:** Reading man flap, Skin plasty, Neoplasm, Reconstructive surgery, Wound closure, Reconstructive technique

## Abstract

**Background:**

The reading man flap is a novel technique in human medicine for the closure of cutaneous circular defects. To the best of our knowledge, no recent clinical studies have described this procedure in small animals.

**Case presentation:**

In this case series, we present four dogs in which neoplasms were reconstructed using the reading man procedure, which is a double-advancement transposition subdermal flap. The reading man flap was applied in wound revision after surgical removal of a neoplasm in two dogs and in the closure following the excision of a neoplasm in another two dogs. Successful tension-free closure of the lesion site was achieved in all four patients. The postoperative period was uneventful in all patients, and there was no flap necrosis or surgical site infection, although surgical site infection preceded in two cases. Minor complications included partial suture dehiscence in one dog and seroma formation in two dogs. Only one dog required a second anesthesia to insert an active drainage system. The follow-up examination of all four dogs revealed no further complications with the reading man flap at time of the latest wound reevaluation conducted by the surgeon.

**Conclusion:**

The reading man flap is a well-vascularized fasciocutaneous flap that provides tension-free closure owing to its asymmetrical Z-plasty. It is a simple-to-use option for the closure of circular skin lesions in dogs.

## Background

The reading man procedure is a novel technique for treating skin lesions in human medicine. It was first published in 2008 by Mutaf et al. for the closure of circular dermal defects. The reading man flap (RMF) is created by combining two skin flaps using additional skin relaxation in the form of an unequal Z-plasty manner [1].

Before starting the surgical procedure, the relaxed skin tension lines for the circular defect must be determined. The central limb of the unequal Z-plasty was placed perpendicular to these lines to obtain better closure. An imaginary tangential line passing through the margin of the circular defect was drawn. The lengths of these central limbs were designed to be 50% greater than the diameter of the circular lesions. Beginning at the free end of this line, another line was drawn at an angle of 60°. The first upper quadrangular flap was designed with the upper pedicle lateral to the defect. Starting from the other end of the central limb, a third imaginary line was drawn at an angle of 45°. The second flap is conceived in a triangular manner, with the vertex at the base of the substance loss [1–4].

**Fig. 1:**
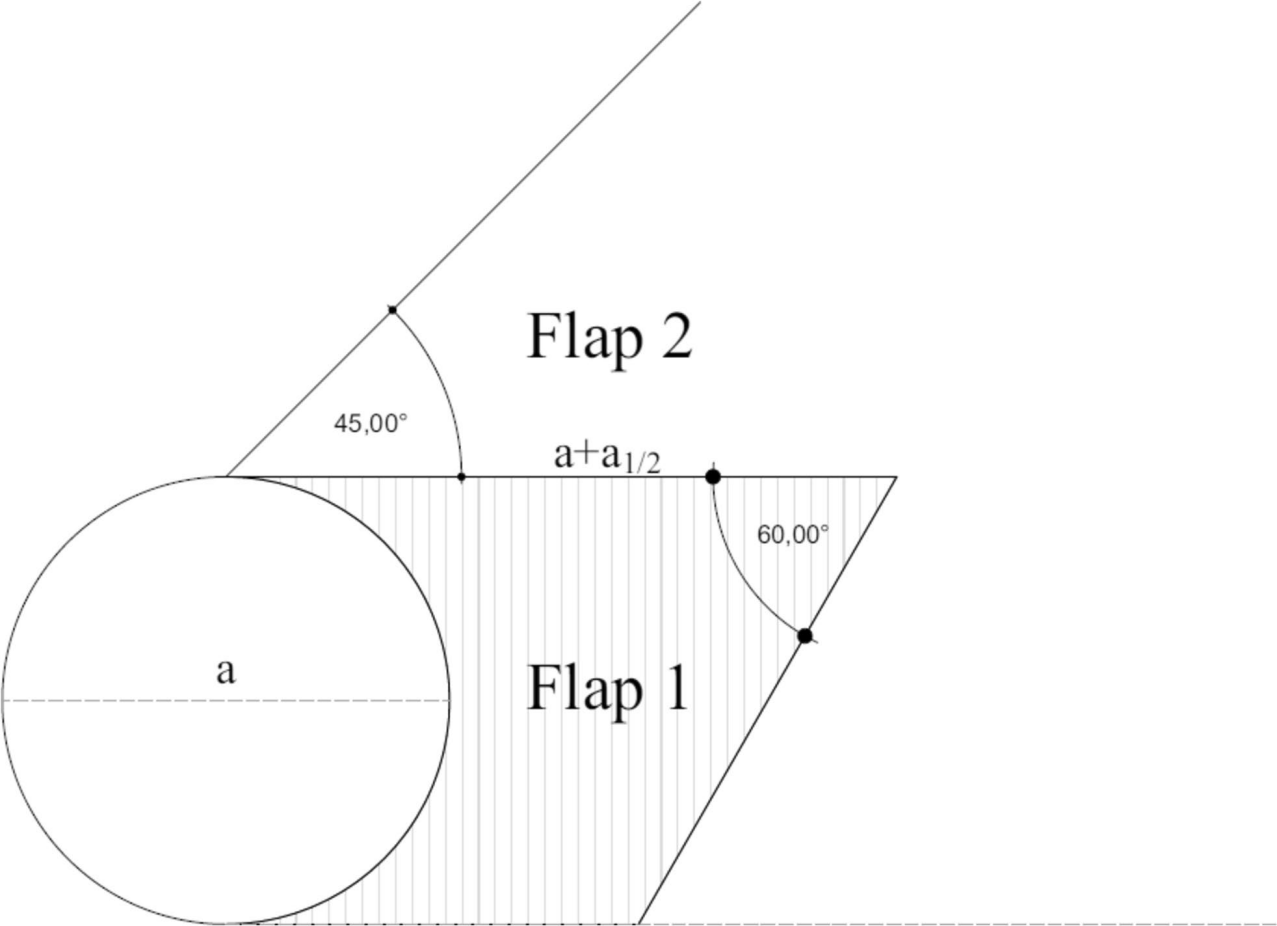
schematic presentation of the RMF

Using this design, two skin flaps of the same length were obtained (Fig. 1). The first flap was transposed into the circular defect area and the second flap covered the donor site of the first flap. Mutaf et al. reported that this procedure could be performed completely either cutaneously or fasciocutaneously, as required. After elevation and movement of the flap, a suction drain was placed beneath the skin flaps, and skin closure was performed in a 2-layer fashion [1–4].

The RMF got its name after its design because it is similar to the silhouette of a man reading a book held in hand [1].

In human medicine, RMF is applied to cover defects following the excision of skin neoplasms located in the fascial area or trunk, as well as for large benign facial skin lesions, such as those in the malar, temporal, or infraorbital regions [1, 2, 4–7].

Several case studies reported its use in the closure of flap donor site defects of the extremities [1]; reconstruction of plantar defects [8]; nasal skin defects [9]; fingertip reconstruction [10]; skin reconstruction after myelomeningocele repair [11]; and pressure sore reconstruction in the sacral, ischial, and trochanteric regions [3].

RMF is a valuable treatment option for circular lesions on different parts of the body in human medicine. It is described for tension-free closure with no to minimal additional healthy skin excision and to prevent the formation of dog-ears and displacement of neighboring mobile anatomic landmarks during fascial reconstruction to keep the symmetry (e.g. deviation of the eye lid or ear) [1, 2, 5, 11].

The use of RMF in humans resulted in good cosmetic and aesthetic results without dog-ear formation, ectropion, or distortion of the surrounding anatomic structures [1, 2, 5, 6, 9, 10, 12]. In addition, in terms of the total scar area and length, the RMF was the one with the most aesthetic outcome [5].

In human medicine, the reading man procedure is an easy and useful alternative for the closure of circular skin defects of different sizes and locations compared with other local flap techniques [1].

The aim of this study was to describe, for the first time, “the reading man procedure”, based on clinical cases in dogs, as a novel technique for the closure of cutaneous defects.

To the best of our knowledge, there have been no recent studies on this procedure in small animals.

## Case presentation

### Case 1

A 10-year-old female Labrador Retriever presented to the University Clinic for Companion Animals of Vienna with a mass on the left elbow. The dog was apathetic for a few days and showed lameness on its left forehand.

General clinical examination was unremarkable. A mast cell tumor was presumed by cytologic examination. At tumor staging, no abnormalities were found by blood work, thoracic radiographs, and abdominal ultrasound.

The 5 × 4 cm tumor cranially to the elbow, was resected with 2 cm margins and a subdermal plexus flap from the distal elbow was performed. A Penrose drain and protective bandage were placed. Histological analysis of the tumor confirmed cytological diagnosis of cutaneous mast cell tumor.

Two days postoperatively, the dog was in clinically good condition, but the wound showed dehiscence. Due to dehiscence, an oral antibiotic, amoxicillin–clavulanic acid (22 mg/kg body weight twice daily) was administered, and a daily change of the protective bandages was performed. Subsequently, the wound became necrotic, and the limb was edematous. Samples were taken from the wound for a bacteriological examination.

Due to a surgical site infection with *Pseudomonas aeruginosa* and *Staphylococcus pseudointermedius*, they were converted to a vacuum-assisted closure (VAC) system, and the antibiotics were changed to marbofloxacin (3 mg/kg body weight once daily orally).

On day 7, after starting the VAC system, primary closure of the granulated wound with a diameter of 6.7 cm in the elbow region was accomplished using a modified reading man procedure.

The lateral wound margin was extended semicircularly to the lateral side of the elbow to mobilize the skin mediodorsally. To reduce the tension at the proximal edge, two skin incisions were made proximally at an angle of approximately 45°. The flap between the two incisions was grafted distally. Closure of the subcutis was achieved with a 3/0 monofilament, resorbable single knots and cutis with a 3/0 monofilament non-absorbable Z-sutures. A Penrose drain was used again.

Postoperatively, a Spica splint was placed and changed daily for the first few days. Immediately after surgery, the flap was vital, with slight dark swelling at the top of the 45° flap. The drainage was highly productive, and generalized erythema was observed in the flap area, but no necrotic areas being noted.

Three days postoperatively, suture dehiscence was observed at the most cranial part of the flap, where the stress due to flexion and extension of the elbow was highest. These areas were treated with second-intention healing adapted to the wound healing phase (e.g. honey or hydrogel) and additional protective bandages.

The wound improved, the Penrose drain could be removed on day 6 postoperatively and 23 days after surgery, complete closure of the wound was achieved.

### Case 2

A 7-year-old intact mixed-breed female was presented to the University Clinic for Companion Animals of Vienna for evaluation of a mass in the left hindlimb. The owner reported that the mass had change in size and shape.

A mass of 10 × 5 cm, pedunculated, of firm consistency, and separated from the underlying musculature, and located laterodistal to the left tibia was detected. Cytological examination of the mass revealed a sarcoma. The distal third of the mass was ulcerated and necrotic.

Tumor staging, including complete blood count, thoracic radiographs, as well as computed tomography scans of thorax and abdomen, revealed no abnormalities.

A grade II canine cutaneous soft tissue sarcoma [13], confirmed by histological examination, was removed with 2 cm margins and primary closure was performed. In the postoperative phase, the wound exhibited mild serosanguinous discharge when the left hindlimb was in motion and tension in the central area of the wound could be observed during the stance phase. Suture dehiscence occurred 15 days postoperatively. *Staphylococcus pseudintermedius* was isolated from the wound exudate by bacteriological examination and an oral cefalexin (20 mg/kg body weight twice daily) was administered postoperatively.

**Fig. 2:**
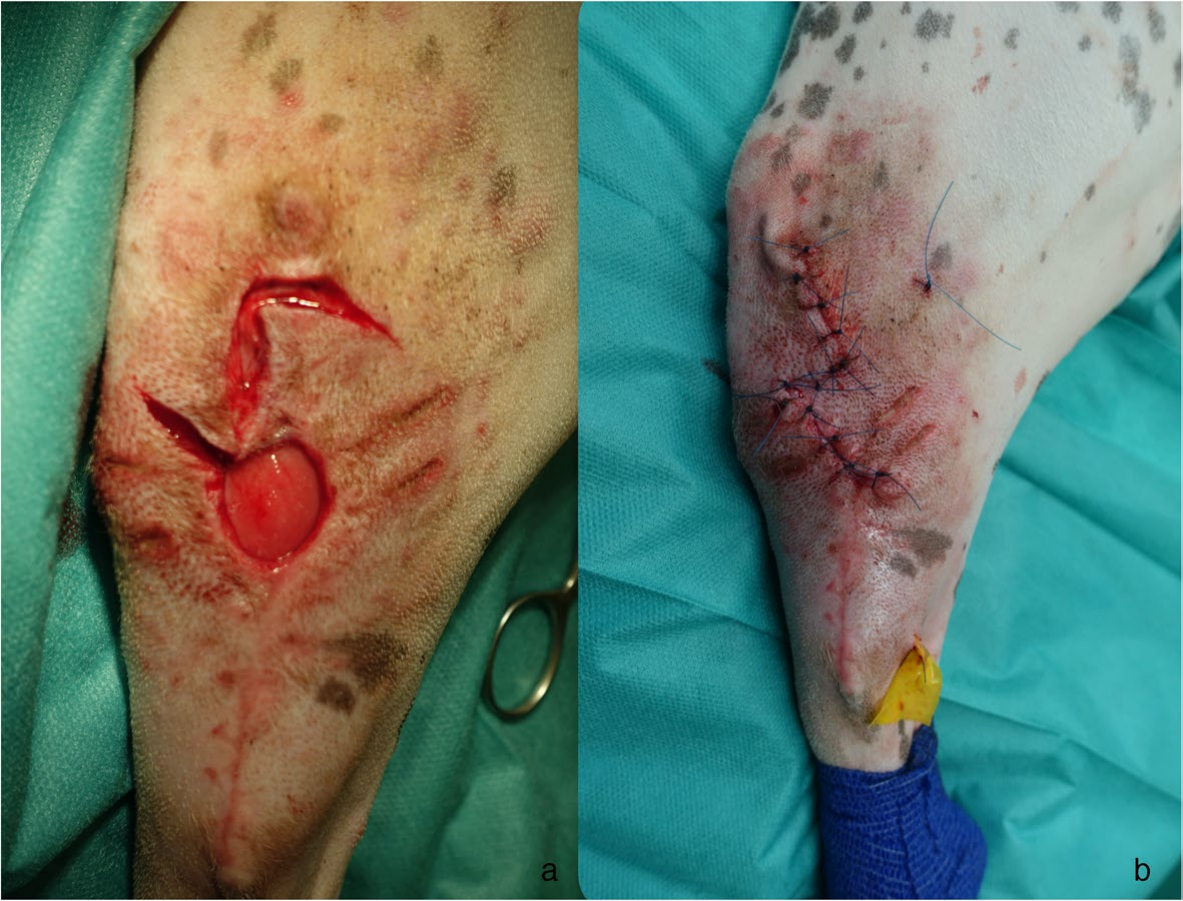
**a**-**b** Case 2; A 7-year-old intact mixed-breed female with a mass, 10 × 5 cm in size, laterodistal to the left tibia. RMF as a reconstructive technique after removing a soft tissue sarcoma

Twenty-two days after the first surgery, the dog underwent skin flap plasty using the reading man procedure. The 3 × 3 cm in size wound was completely granulated (Fig. 2a). Closure of the subcutis was performed with a 3/0 monofilament, resorbable suture material and cutis with a 3/0 monofilament non-absorbable suture material, both in a simple interrupted pattern.

A tension-free skin closure was achieved, and a Penrose drain was placed due to surgical site infection (Fig. 2b). Antibiotics were changed to oral amoxicillin–clavulanic acid (22 mg/kg body weight twice daily) for 20 days.

Postoperative recovery was uneventful, and the dog was discharged one day postoperatively. On day 5, the Penrose drain was removed.

Follow-up examination 20 days after surgery showed complete wound closure.

### Case 3

A 10-year-old intact male Golden Retriever presented to the University Clinic for Companion Animals of Vienna with two masses in the right shoulder and cranial to the prepuce. A cytological examination confirmed the presence of a mast cell tumor. Clinical and blood examination results were unremarkable. No signs of metastasis were observed on thoracic radiography or abdominal ultrasound.

**Fig. 3:**
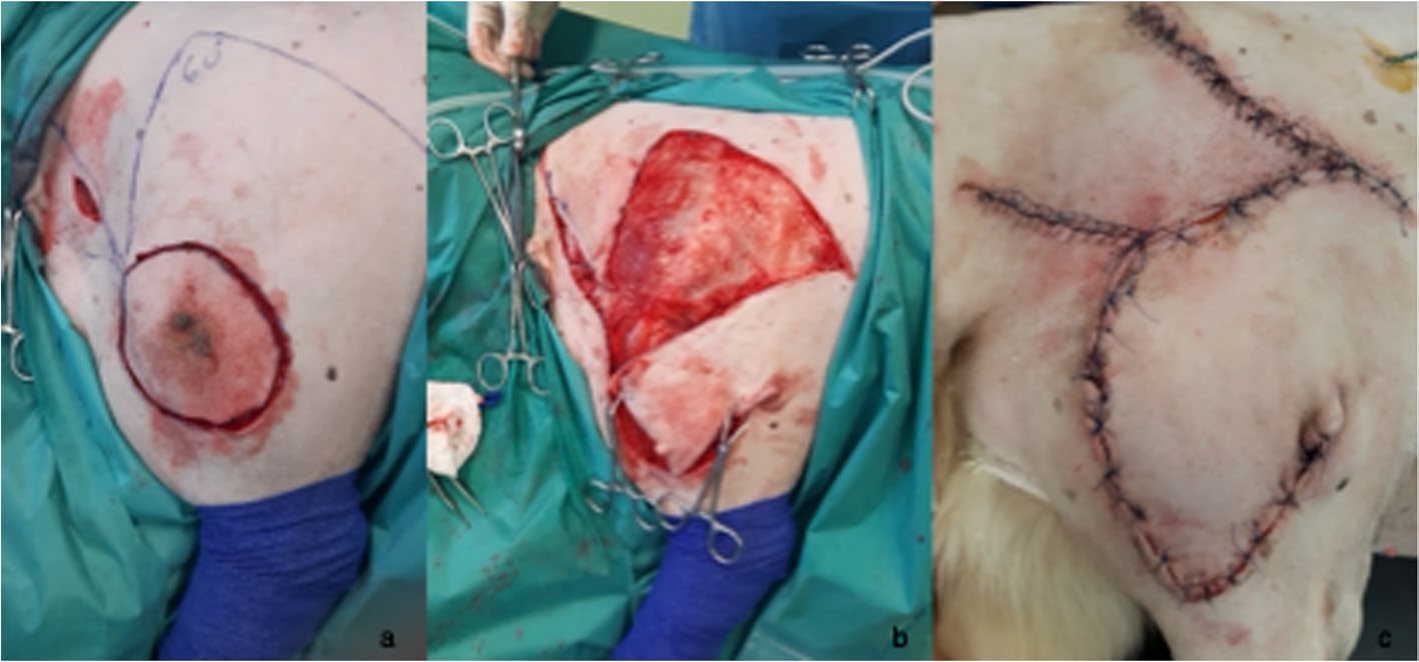
**a**-**c** Case 3: A 10-year-old intact male Golden Retriever with a mass at the right shoulder. RMF as a reconstructive technique after removing a mast cell tumor

The shoulder neoplasm, 9 × 7 cm in size, was excised circularly with a margin of 2 cm, and the underlying fascia was removed. The reading man procedure was performed with the central limb directed dorsally at the cranial margin of the circular area, and the second flap was created craniodorsally at a 45° angle. The caudodorsal flap was transposed distally to cover the lesion area and a cranial triangular flap was used to cover the donor site (Fig. 3a-c).

An active drain was placed for two days. Closure of the subcutis was done with a 4/0 monofilament, resorbable suture material in a simple continuous pattern and cutis with a 3/0 monofilament non-absorbable suture material in a Z-suture technique. The prescapular lymph node were surgically removed, and the second mass was excised by using an H-skin plasty technique [14].

Postoperatively, the dog received cephazolin (22 mg/kg body weight thrice daily intravenously) for 6 days and was discharged without any complications related to the RMF 7 days after surgery. Examination of the wound revealed no intraoperative bacterial growth. The pathohistological examination determined a mast cell tumor in the shoulder region but lymph node samples showed no evidence of metastasis.

Thirteen days after tumor resection, the dog presented with seromas in both regions. Unfortunately, for reasons unknown, the active drainage system failed. Consequently, the developing seroma has been addressed by implementing a Penrose drain in the RMF. The Penrose drain remained in place for a period of 14 days due to its consistently high fluid output. No signs of surgical site infection, necrosis, or other abnormalities were observed.

At follow-up (26 months later), the owner described no further complications with the RMF.

### Case 4

An 11-year-old female spayed Border Collie was evaluated for a neoplasm of the right abdominal trunk.

General clinical examination results were unremarkable. The mass, presumed by cytologic examination as a soft tissue sarcoma, was craniolateral to the right proximal femur, approximately 7 × 4 cm in size, round, immobile, and firm.

Tumor staging revealed no abnormalities on thoracic radiography, abdominal ultrasonography, or blood examinations.

The soft tissue sarcoma was removed using a circular cut with 2.5 cm margins and 3 cm depth with excision of the fascia lata, and dorsally directed RMF was performed. Suturing of the subcutis was performed with 3/0 monofilament, resorbable suture material in a simple continuous pattern and cutis with a 3/0 monofilament non-absorbable suture material in a Z-suture technique. The dog was discharged one day postoperatively with cefazolin (20 mg/kg twice daily). Pathohistological evaluation of the tumor confirmed cytological diagnosis of a soft tissue sarcoma.

An active drainage was placed and removed three days postoperatively by the referral veterinarian.

On follow-up 7 days after discharge a seroma in the distal right limb was observed, but without any consequence on the RMF.

## Discussion

Tension-free closure of the skin after the removal of a cutaneous circular neoplasm is often challenging. In human medicine, RMF is increasingly used for skin flap surgery in such wounds [1]. To date, there is no evidence of the clinical application of this flap in small animal surgery. An anecdotal description of the flap appeared in a veterinary textbook [15].

In the present study, we report a case series of four clinical patients that had reconstruction of cutaneous defects following the excision of various neoplasms using the reading man procedure, a double-advancement transposition subdermal flap named after its appearance [1, 6].

The common Z-plasty is an effective technique for relieving tension in two directions by making additional skin available. Two triangular skin flaps were transposed from the common central limb to allow relaxation along it. All legs in Z-plasty must be of equal length, starting with the central limb and directed along the line of tension. The two arms extending from the ends of the central limb were inclined at an angle of approximately 60° (range, 30–90°). In veterinary medicine, this flap design is described for use along the cicatrix or to facilitate the closure of nearby wounds, but not for the removal and coverage of a circular tumor or rounded skin lesions [14].

The RMF is a combination of two skin flaps drafted in an irregular Z-plasty manner for circular lesions located in different parts of the body [1]. This design maximizes the extent of tissue relaxation, and the closure of a rounded defect can be achieved with significantly decreased tension. Tissue is harvested from two different directions, distributing strain and causing less tissue distortion and displacement of neighboring mobile anatomical landmarks, especially in facial reconstruction [1–3]. Therefore, during this procedure, it is not necessary to remove additional healthy tissue; instead, it uses the advantages of two well-vascularized fasciocutaneous flaps which provide durable coverage [2, 5, 11].

In human medicine, the reading man procedure is performed for the excision of circular skin cancers located in the fascial area or trunk, as well as for large benign facial skin lesions, such as those in the malar or infraorbital region [1, 2, 5–7].

The RMF is restricted to circular skin defects; however, a human study reported a modified RMF to cover rectangular infraorbital and malar skin lesions [1, 16]. One limitation of RMF is that the defects are localized in areas with poorly distensible skin, such as the scalp [4].

In this case series, the RMF was applied in wound revision after the surgical removal of neoplasms and for coverage of a circular defect following the excision of a neoplasm.

In human medicine, almost all authors refer to good cosmetic and aesthetic results and no reports of postoperative infection, recurrence rate of tumor growth, or necessitate further surgical investigations [1–3, 5, 6, 9, 10, 12]. In addition, a successful tension-free closure was achieved in all four patients without dog-ear formation. There was no flap necrosis or surgical site infection in none of our patients, although surgical site infection preceded in cases 1 and 2.

However, there was a human case series with 32 patients in which < 5% local necrosis occurred in one flap and delayed healing occurred in five cases [12].

Another study reported minimal tip necrosis in one of seven patients; however, healing was uneventful without any complications [11]. Among the four dogs in this report, one developed partial suture dehiscence, which was addressed with second-intention healing and additional protective bandages. We suspect that this was due to the increased movement in the elbow region and the accompanying stress on the wound and sutures.

Two other dogs developed postoperative seroma despite drainage (Case 3 and 4). One of them had to be treated with the placement of a new active drain in a second anesthesia (Case 3). The second dog was treated by a referral veterinarian; however, a follow-up call reported healing without further treatments (Case 4). It’s worth noting that the first one experienced a significant seroma due to several contributing factors. These included the large size of the dog (Golden Retriever), the extensive size of the flap utilized, the proximity of the flap site to the shoulder joint, which allowed for substantial mobility, and the fact that restriction by the owners was limited. In contrast, case 4 had a minor seroma that had no surgical consequences. Seroma development in this case could be attributed to the mobile nature of the skin in the abdominal trunk area, where movement is more pronounced.

Thus, two patients underscore the importance of considering the placement and duration of drainage after surgery, and perhaps it should be discussed whether to leave them in situ longer or using additional drainage to prevent the development of a seroma. We may also use active drainage, as opposed to Penrose drains, to support discharge and to prevent potential infections in these cases. A larger number of cases and prospective studies are required to confirm this assessment.

The main limitation of this study includes its retrospective nature, the inclusion of diverse dog breed, the absence of a control group utilizing alternative flap techniques, variations in size and surgical sites of the tumor, and the diversity tumor types.

In comparison to classical Z-plasty, which is useful for the closure of nearby wounds but not for circular lesions, RMF seems to be an excellent alternative for tension-free skin closure after the removal of circular neoplasms in dogs by exploiting the advantages of extra tissue relaxation provided by asymmetrical Z-plasty [9, 14].

In conclusion, the RMF is a well-vascularized fasciocutaneous flap that provides tension-free closure owing to its unequal Z-plasty. In our four patients, favorable outcomes with RMF were achieved, without any major complications. In 2/4 dogs seroma formation postoperatively occurred which could be preventively addressed by active drainage.

This flap is a useful alternative for the coverage of cutaneous circular defects following the excision of neoplasms or rounded skin lesions in dogs.

## Data Availability

All data generated or analyzed during this study are included in the published article.

## Abbreviations

RMF: Reading Man Flap
VAC: Vacuum-assisted closure
